# Outcomes of Microsurgical, Endovascular, Multimodal, and Radiosurgical Management of Spinal Intramedullary Arteriovenous Malformations: A Systematic Review and Exploratory Meta-Analysis

**DOI:** 10.1016/j.bas.2026.106654

**Published:** 2026-08-18

**Authors:** Badr Hafiz, Thamer Alsharif, Fahad Okal, Faisal Sukkar, Yazeed Alotaibi, Lamair Albakri, Khalid Bajunaid, Saleh Baeesa

**Affiliations:** a Department of Neurosciences, King Faisal Specialist Hospital and Research Centre, Jeddah, Saudi Arabia; b Neurosurgery Department, King Abdulaziz Specialist Hospital, Taif, Saudi Arabia; c Department of Neuroscience, King Abdulaziz Medical City, National Guard Health Affairs, Jeddah, Saudi Arabia; d Department of Neurosurgery, Dr. Sulaiman Al Habib Medical Group Holding Company, Jeddah, Saudi Arabia; e Neurosurgery Department, Al Noor Specialist Hospital, Makkah Cluster, Saudi Arabia; f Department of Surgery, College of Medicine, University of Jeddah, Jeddah, Saudi Arabia; g Faculty of Medicine, Alfaisal University, Riyadh, Saudi Arabia

**Keywords:** Arteriovenous malformations, Endovascular embolization, Intramedullary arteriovenous malformations, Microsurgical resection, Radiosurgery, Spinal arteriovenous malformations

## Abstract

**Introduction:**

Spinal intramedullary arteriovenous malformations (Si-AVMs) are rare lesions determined by angioarchitecture, location, clinical presentation, and procedural intent, making comparisons between modalities vulnerable to confounding by indication.

**Research question:**

To characterize radiographic, functional, and safety outcomes after microsurgical, endovascular, combined, and radiosurgical management of Takai Type II/III Si-AVMs while distinguishing complete treatment, planned partial embolization, residual disease, recanalization, and multimodality care.

**Materials and methods:**

A PRISMA-guided systematic review and exploratory meta-analysis of studies published from 2000 to 2024 included seven retrospective studies comprising up to 672 Type II/III or nidus-type lesions. Treatment pathways and embolization intent were analyzed separately. Combined-treatment patients were retained as a distinct group unless outcomes were disaggregated. Because outcome definitions were heterogeneous, pooled estimates were considered descriptive and hypothesis-generating.

**Results:**

Microsurgical series generally reported higher complete angiographic obliteration than embolization-only series; however, lesion selection and planned multimodality treatment precluded causal comparison. In the largest cohort, 68/258 (26.4%) embolization-only patients achieved complete obliteration. Recanalization occurred in 15/30 (50.0%) lesions initially considered completely obliterated and in 73/219 (33.3%) embolized lesions. After partial embolization, lesion proliferation and de novo aneurysm formation each occurred in 22/191 (11.5%) patients. Permanent neurological deterioration occurred more often after microsurgery than embolization (16.1% vs. 5.6%).

**Discussion and conclusion:**

The available evidence supports lesion-specific, complementary use of microsurgery, embolization, combined treatment, and radiosurgery rather than modality-wide superiority. Microsurgery may provide durable obliteration in selected lesions, whereas embolization may be curative, staged, neoadjuvant, or palliative. Radiosurgery remains a niche option for selected, inaccessible lesions. All conclusions are hypothesis-generating.

## Highlights

• Systematic review and exploratory meta-analysis of Type II/III spinal intramedullary AVMs (Takai classification).
• Microsurgery, embolization, combined treatment, and radiosurgery have complementary roles based on lesion characteristics.
• Complete angiographic obliteration was generally higher after microsurgery, but comparisons were limited by selection bias.
• Embolization may be curative, staged, neoadjuvant, or palliative depending on treatment intent and lesion anatomy.
• Current evidence is hypothesis-generating because available studies are retrospective and methodologically heterogeneous.

## 1 Introduction

Spinal intramedullary arteriovenous malformations (Si-AVMs) are rare vascular lesions characterized by abnormal arterial–venous connections within the spinal cord, accounting for 3–4% of space-occupying spinal cord lesions (Endo et al., 2016). Despite their rarity, they are associated with significant morbidity due to spinal cord ischemia, mass effects, and recurrent hemorrhage (Endo et al., 2016; Akgun et al., 2019).

The classification of spinal vascular lesions has evolved over time. Early histopathological descriptions by Bergstrand et al. (1964) used terms such as *angioma racemosum*. Rosenblum et al. (1987) (1987) later introduced a clinically oriented system distinguishing dural AVFs from intradural AVMs and subclassifying intramedullary lesions into glomus (compact) and juvenile (diffuse) types. Subsequently, Spetzler et al. (2002) (2002) proposed a pathophysiology- and imaging-based classification incorporating aneurysms, AVFs, and AVMs, aligning the terminology with surgical decision-making.

Despite these advances, heterogeneity in reported outcomes persists. Takai et al. (Takai, 2017) (2017) proposed a unified classification based on anatomy and morphology, dividing spinal arteriovenous shunts into five types: Type I (dural AVF), Type II (glomus AVM), Type III (juvenile AVM), Type IV (perimedullary AVF), and Type V (extradural AVF). This system improves the consistency of diagnosis, research, and treatment planning (Kona et al., 2021).

**Fig. 1:**
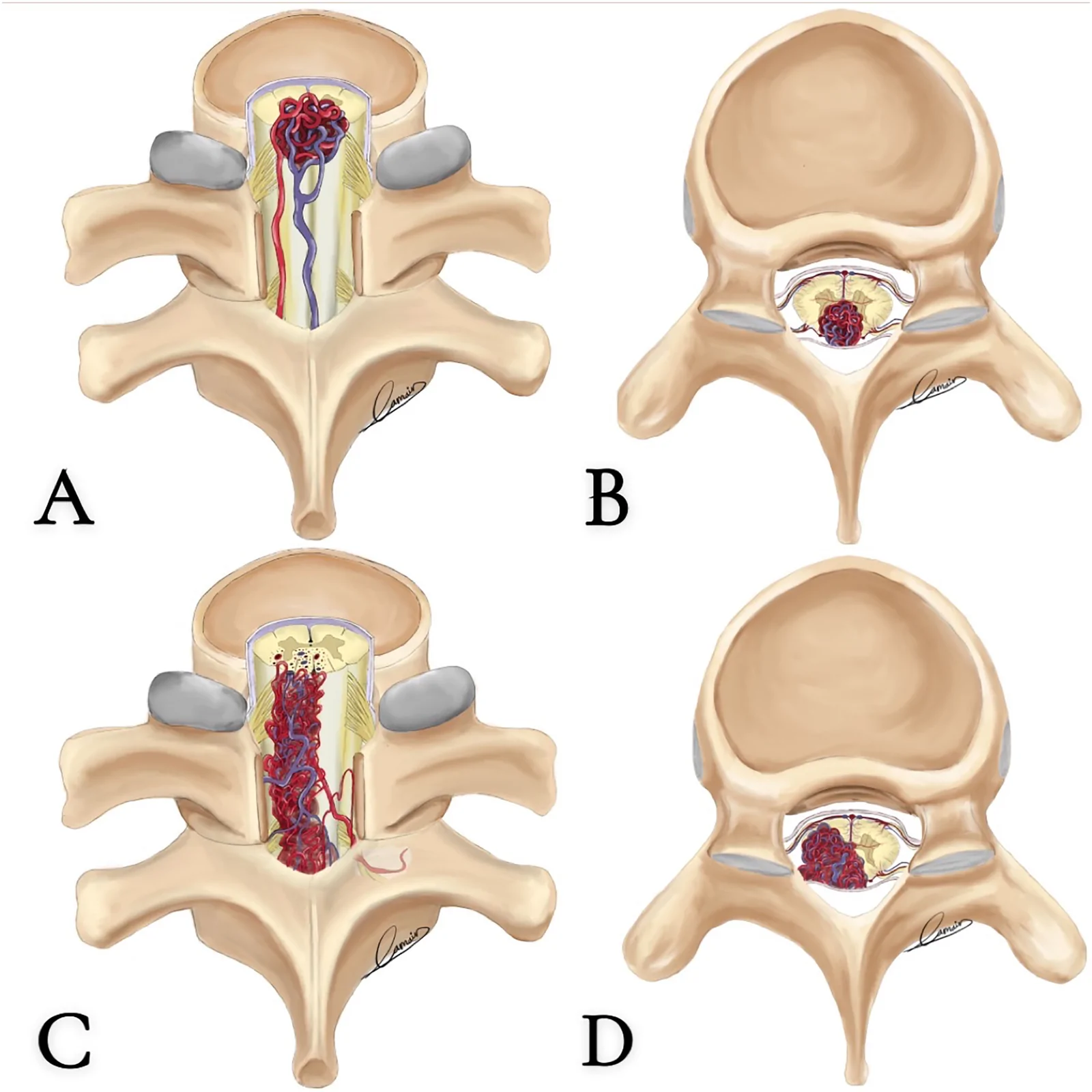
Diagram illustrating Type II and Type III Si-AVMs. Type II Si-AVMs are shown in coronal (A) and axial (B) views with a compact intramedullary nidus supplied by radiculomedullary or pial arteries and draining into the intramedullary and perimedullary veins. Type III Si-AVMs are shown in coronal (C) and axial (D) views with a diffuse nidus that may involve multiple tissue planes, multiple feeding arteries, and extensive venous drainage.

Type II and III Si-AVMs (Fig. 1A–D) remain among the most challenging spinal vascular lesions because of their intramedullary location, multiple feeders, and complex venous drainage (Takai, 2017; Mamaril-Davis et al., 2023). Type II glomus lesions are compact, whereas Type III juvenile or metameric lesions are diffuse and may traverse multiple tissue planes (Takai, 2017; Gross and Du, 2013). Their architecture, segmental location, feeder pattern, venous drainage, and relationship to functional cord tissue determine whether microsurgery, embolization, combined treatment, radiosurgery, or observation is feasible (Mamaril et al., 2023; Wilson et al., 2012; Velat et al., 2012).

Treatment options for Si-AVMs include microsurgical resection, endovascular embolization, stereotactic radiosurgery (SRS), and multimodal combinations. These approaches do not uniformly compete with standalone treatments. Microsurgery can achieve complete resection in selected compact lesions but carries the risk of neurological morbidity (Velat et al., 2012). Embolization may be used with curative intent, for neoadjuvant flow reduction, as staged shunt reduction, to target an aneurysm or other high-risk compartment, or as palliation; planned partial embolization should not automatically be classified as treatment failure (Kona et al., 2021; Wilson et al., 2012; Yu et al., 2021; Krings et al., 2009). SRS has a limited, selective role in deep or surgically inaccessible lesions, but the available evidence consists mainly of small heterogeneous series (Zhan et al., 2019). Therefore, treatment selection must be individualized according to the lesion architecture, location, presentation, treatment objective, and anticipated procedural risk.

This systematic review evaluated treatment pathways rather than assuming that surgery and embolization are mutually exclusive. The primary objective was to characterize radiographic durability after documented complete treatment and the evolution of deliberately or unintentionally residual lesions. The secondary objectives were to summarize functional outcomes, complications, mortality, rehemorrhage, crossover, treatment intent, subtype, location, and embolic agent reporting. Formal comparative claims were limited to data reported by the source studies with compatible definitions.

## 2 Methods

A systematic review was performed according to the Preferred Reporting Items for Systematic Reviews and Meta-Analyses (PRISMA) 2020 guidelines to investigate the outcomes of Si-AVM treatment (Parums, 2021). This protocol was prospectively registered in the International Prospective Register of Systematic Reviews (CRD420251142499), and institutional review board approval was not required for this study.

### 2.1 Search strategy

Major databases (PubMed, MEDLINE, Embase, Web of Science, and Scopus) were searched from January 2000 to December 2024. Boolean operators (AND/OR) were used to combine terms associated with the Si-AVM, intervention, lesion type and outcomes (Table 1). To find more pertinent articles that were not found during the first search, backward and forward citation tracking of the included studies and key reviews was performed. Only English-language studies were included, which represents a potential limitation, given that meaningful institutional series from non-English sources may have been excluded.

**Table 1:** Search terms and strategy.

Database	Search Terms/Strategy
PubMed	(“spinal arteriovenous malformation"[MeSH] OR "spinal AVM” OR "intramedullary AVM” OR "glomus AVM” OR "juvenile AVM”) AND (“microsurgery” OR "surgical resection” OR "endovascular embolization” OR "stereotactic radiosurgery” OR "Gamma Knife” OR "CyberKnife”) AND (“obliteration” OR "outcome” OR "recurrence” OR "complications”) Filters applied: Humans, English, 2000–2024
MEDLINE	exp *spinal arteriovenous malformations/*OR (“spinal arteriovenous malformation” OR "intramedullary AVM” OR "glomus AVM” OR "juvenile AVM”). mp. AND (exp *microsurgery/*OR *embolization, therapeutic/*OR "surgical resection”. mp. OR "stereotactic radiosurgery.” mp. OR "Gamma Knife".mp. OR "CyberKnife".mp.) AND (“outcome”. mp. OR "recurrence".mp. OR "complication".mp. OR *treatment outcome/*OR *embolization, therapeutic/*) Limits: Humans, English language, 2000–2024
Embase	('spinal arteriovenous malformation'/exp OR 'intramedullary avm' OR 'glomus avm' OR 'juvenile avm') AND ('microsurgery'/exp OR 'surgical resection' OR 'endovascular embolization' OR 'stereotactic radiosurgery' OR 'gamma knife' OR 'cyberknife') AND ('outcome' OR 'recurrence' OR 'complication' OR 'obliteration') Filters: Human, English, 2000–2024
Web of Science	TS = (“spinal arteriovenous malformation” OR "intramedullary AVM” OR "glomus AVM” OR "juvenile AVM”) AND TS = (“microsurgery” OR "endovascular embolization” OR "stereotactic radiosurgery” OR "Gamma Knife” OR "CyberKnife”) AND TS = (“outcome” OR "recurrence” OR "complication” OR "obliteration”) Timespan: 2000–2024; Language: English
Scopus	TITLE-ABS-KEY(“spinal arteriovenous malformation” OR "intramedullary AVM” OR "glomus AVM” OR "juvenile AVM”) AND TITLE-ABS-KEY(“microsurgery” OR "surgical resection” OR "endovascular embolization” OR "stereotactic radiosurgery” OR "Gamma Knife” OR "CyberKnife”) AND TITLE-ABS-KEY(“outcome” OR "recurrence” OR "complication” OR "obliteration”) Limit to: English, 2000–2024

### 2.2 Study selection and data extraction

Duplicate records were eliminated using EndNote (Clarivate Analytics), and the remaining articles were screened using Rayyan (https://rayyan.qcri.org/). Two reviewers (B.H. and T.A.) independently screened the titles and abstracts, followed by a full-text article evaluation based on the pre-specified inclusion and exclusion criteria (Table 2). The primary investigator (S.B.) was consulted to resolve any disagreements between the reviewers. The final analysis included original studies that fulfilled the inclusion criteria. Data extraction was independently performed by the same reviewers. The extracted variables included demographics, neurological status, AVM characteristics, treatment details, and outcomes (recurrence, obliteration, complications, re-hemorrhage, and mortality).

**Table 2:** Inclusion and exclusion criteria.

Characteristics	Inclusion Criteria	Exclusion Criteria
**Language**	English-language publications	Non-English publications
**Time Period**	January 2000–December 2024	NA
**Study Design**	Randomized controlled trials (RCTs); non-randomized comparative studies; prospective or retrospective cohort studies; case series with ≥3 patients	Single case reports (n = 1) or 2-patient reports; editorials; letters; narrative reviews; conference abstracts; duplicate datasets
**Population**	Patients with spinal intramedullary or nidus-type AVMs classified as Takai Type II or III; mixed-age series were eligible when relevant lesion-specific outcomes were extractable.	Studies of Takai Types I, IV, or V; intracranial AVMs; or mixed spinal vascular cohorts without extractable Type II/III or nidus-type data.
**Intervention**	Stereotactic radiosurgery (Gamma Knife, CyberKnife, LINAC-based), endovascular embolization (n-BCA/NBCA, Onyx, coils) or microsurgical resection	NA
**Comparator**	Any of the above interventions were compared directly or indirectly; combined treatment was retained as a separate pathway when reported.	NA
**Outcomes**	Complete obliteration, residual disease, recurrence after documented cure, recanalization, lesion progression or proliferation, functional outcome, rehemorrhage, mortality, and standardized treatment-related complications.	Articles without extractable data relevant to predetermined outcomes
**Context**	Studies involving human subjects with Si-AVMs evaluated using the Takai classification	Non-human or experimental animal studies

Before synthesis, treatment exposure was classified as (1) embolization alone with curative intent, (2) planned partial or targeted embolization, including shunt reduction or palliative treatment of aneurysms or other high-risk compartments, (3) microsurgery alone, (4) planned neoadjuvant or staged embolization followed by surgery, (5) adjuvant embolization after surgery, (6) radiosurgery alone or as an adjunct, and (7) conservative management. Patients receiving combined treatment were retained as a separate pathway unless a study provided disaggregated outcome data. Unplanned crossover was defined as subsequent surgery required after an initial embolization strategy and was recorded only when the original report distinguished it from planned multimodality care. Data were also sought by Takai subtype, compact versus diffuse or metameric architecture, spinal level, embolic agent, complication type, timing, and permanence.

### 2.3 Quality assessment and outcomes measures

Given that all included studies were retrospective observational cohorts or case series rather than randomized controlled trials, the methodological quality was assessed using the Newcastle-Ottawa Scale (NOS) for non-randomized studies. The NOS evaluates three domains: selection of cohorts, comparability of cohorts, and ascertainment of outcomes. A high-quality study was defined as a score of ≥7 out of 9 points. The RoB 2 tool was not used because it is designed for randomized trials (Minozzi et al., 2020), and the CASP checklist was not used because it is primarily intended for qualitative evidence appraisal (Long et al., 2020); neither tool was appropriate for the included nonrandomized evidence. Discrepancies were resolved by consensus [Table 3]. presents the NOS scores for each included study.

**Table 3:** Newcastle-Ottawa Scale quality assessment.

Author, Year [Reference]	Selection (Max 4)	Comparability (Max 2)	Outcome (Max 3)	Total Score (Max 9)
Rangel-Castilla et al., 2014 (Rangel- et al., 2014)	4	1	3	8
Lee et al., 2014 (Lee et al., 2014)	4	1	3	8
Boström et al., 2009 (Boström et al., 2009)	3	1	3	7
Lu et al., 2024 (Lu et al., 2024)	4	1	3	8
Yu et al., 2021 (Yu et al., 2021)	4	2	3	9
Ushikoshi et al., 2000 (Ushikoshi et al., 2000)	3	0	2	5
Lizana et al., 2022 (Lizana et al., 2022)	3	0	2	5

Radiographic endpoints were defined a priori as follows: complete obliteration required no residual intradural arteriovenous shunt on digital subtraction angiography (DSA) or explicit angiographic cure on magnetic resonance angiography when DSA was unavailable, and the source study used that definition. Residual disease denotes a visible shunt immediately after intentionally or unintentionally incomplete treatment. Recurrence was defined as the reappearance of a shunt after documented complete obliteration. Recanalization specifically refers to the reperfusion of a previously embolized and angiographically occluded shunt. Progression or proliferation denoted the enlargement or new angioarchitectural features of a known residual lesion. Planned partial embolization was not coded as treatment failure; outcomes were separated, when possible, into shunt reduction and palliative or high-risk-target strategies. Functional outcomes were extracted using the modified McCormick, modified Aminoff-Logue, ASIA Impairment, or modified Rankin scales. Complications were categorized as hemorrhagic, ischemic, thromboembolic, surgical, cerebrospinal fluid-related, or radiation-related. Acute versus delayed and transient versus permanent statuses were recorded when available.

### 2.4 Statistical analysis

Analyses were performed using Review Manager 5.4.1 (Cochrane Collaboration) and were specified as exploratory because all included reports were retrospective and treatment assignment was non-random. Random-effects models were used only when at least three studies reported sufficiently comparable endpoints and denominators. Combined-treatment patients were not pooled with surgery-alone or embolization-alone groups unless the source study separated them into different groups. Study-level proportion plots were retained as descriptive displays and were not interpreted as direct causal comparison. Risk ratios were not reported when compatible 2 × 2 data were unavailable. Heterogeneity was assessed using the I2 statistic and Cochran Q test when formal pooling was appropriate. With only seven heterogeneous studies, a formal funnel plot or regression-based assessment of publication bias was not performed. Subtypes, location, treatment intent, embolic agent, and crossover analyses were presented narratively when fewer than three comparable datasets were available.

## 3 Results

**Fig. 2:**
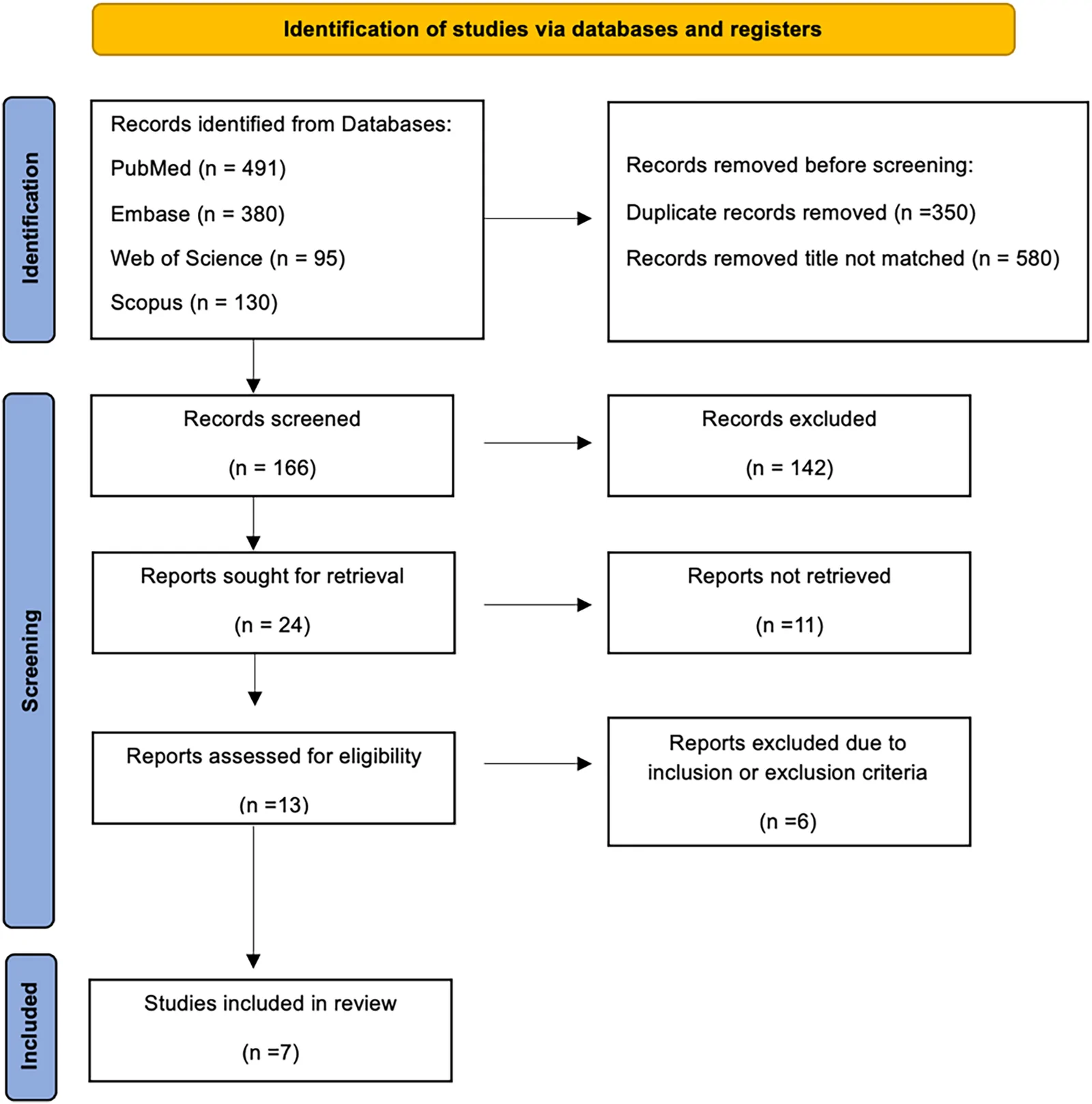
PRISMA flowchart.

A comprehensive database search and manual reference screening identified 1096 records for review. A total of 166 records were screened after 350 duplicates and 580 records that did not meet the inclusion criteria based on their titles were excluded from the study. After reviewing the titles and abstracts, 142 studies were eliminated, leaving 24 remaining reports. Studies such as single case reports, conference abstracts, editorials, and those addressing non-intramedullary shunts (dural, perimedullary, or extradural fistulas) were excluded. To maintain analytical clarity, reports combining Type II and III Si-AVMs with other AVM types without separate data and studies with fewer than three patients were excluded. The remaining 13 full-text articles were evaluated, and six studies were eliminated after a thorough review. Seven studies were included in the qualitative synthesis and meta-analysis. The selection process is summarized in the PRISMA flow diagram shown in [Fig. 2].

### 3.1 Study characteristics

Seven retrospective reports published between 2000 and 2024 contributed to 672 Type II/III or nidus-type observations. Because Yu and Lu's reports arose from large referral networks and individual-level identifiers were unavailable, cohort overlap could not be excluded. The study-level denominators varied by endpoint. Lee et al. included 44 spinal cord AVMs, of which 26 were nidus-type; Yu et al. (Yu et al., 2021)included 548 shunts (463 treated), of which 348 were nidus-type; and Lu et al. (Lu et al., 2024)included 240 cervical shunts with 199 nidus-type lesions used in present lesion-specific summary. Other series included 4-60 relevant lesions. Treatment pathways included embolization alone, surgery alone, planned combined therapy, adjunctive radiation, and conservative management. Table 4A, Table 4BA and 4B together comprise the complete study-characteristics table.

**Table 4A:** Study characteristics, lesion architecture, treatment pathways, and baseline characteristics.

Study	Design and Relevant Sample	Architecture and Location	Treatment Pathway and Intent	Baseline Status	Follow-up
Rangel-Castilla et al., 2014 (Rangel- et al., 2014)	Retrospective single-center series; 110 spinal vascular malformations; AVM outcome denominators up to 66	Approximately 60 relevant intramedullary, conus or metameric lesions; mostly thoracic (61%)	Microsurgery was predominant (86%), followed by perioperative embolization (42%), embolization alone (13%), and radiosurgery (one case). Combined outcomes were not separated.	Mean McCormick approximately 2.5 ± 0.9	Mean 30.5 months (range 1-205)
Lee et al., 2014 (Lee et al., 2014)	Multicenter retrospective cohort; 44 AVMs total; 26 nidus-type	Cervical 27%, upper thoracic 19%, lower thoracic 54%	Nidus subgroup: embolization 18; surgery 4; planned combined 2; conservative 2	Severe deficit at onset in 7/26 (27%)	Mean 13.8 years (median 12.5; up to 34 years)
Boström et al., 2009 (Boström et al., 2009)	Retrospective surgical series; 20 Type II glomus AVMs	Cervical 6, thoracic 6, conus 8	Microsurgery; 7/20 patients received preoperative embolization. Adjunctive versus surgery-alone outcomes were not separated.	McCormick I 7, II 4, III 6, IV 3	6-185 months (median approximately 59)
Lu et al., 2024 (Lu et al., 2024)	Retrospective cervical cohort; 240 shunts total; 199 nidus-type	All cervical: C1-2 15%, C3-5 61%, C6-7 24%	Nidus subset: embolization 120 and microsurgery 79; any combined sequence not separately reported	Baseline mALS median 2 (range 0-5)	Median 32.6 months (IQR 15.6-50.7)
Yu et al., 2021 (Yu et al., 2021)	Multicenter cohort; 548 shunts, 463 treated; 348 nidus-type	All types pooled: cervical 37.3%, thoracic 34.7%, below T9 28.0%; location-specific treatment outcomes unavailable	Embolization only 258; microsurgery only 122; planned combined 83; unplanned crossover not reported	Baseline mALS median 3 (IQR 1-6)	Median 45.1 months (IQR 25.0-70.3)
Ushikoshi et al., 2000 (Ushikoshi et al., 2000)	Retrospective single-center series; 15 intramedullary AVMs	Cervical, thoracic and lumbar; not individually stratified	Embolization 5; surgery 10; radiation adjunct in 4. The sequence and intent are incompletely separated.	Nine hemorrhagic acute myelopathies; six progressive myelopathies	6 months-17 years (mean 7.5 years)
Lizana et al., 2022 (Lizana et al., 2022)	Single-center case series; four mixed-age cases	Two compact and two diffuse; thoracic 1, conus 3	Three embolization-only; one staged embolization, partial surgery and additional embolization	Significant deficits at presentation; mRS 3-4 in several cases	1-5 years

**Table 4B:** Clinical outcomes, radiographic outcomes, complications, and study limitations.

Study	Follow-up Functional Status	Complete Obliteration	Follow-up Radiographic Status	Complications	Key Interpretive Limitation
Rangel-Castilla et al., 2014 (Rangel- et al., 2014)	57/66 (86.4%) functionally independent	50/66 (75.7%)	10/66 (15.2%) residual or recurrent; endpoints not separated	20/66 (30.3%); type, timing and permanence incompletely standardized	Outcome denominator differed from lesion-location subgroup; combined pathway not disaggregated
Lee et al., 2014 (Lee et al., 2014)	Improved 8/26, stable 12/26, deteriorated 6/26	7/26 (27%) overall; 17% embolization vs 67% surgery/combined as reported	Rebleeding 4.8%/year untreated, 2.9%/year partial, 0 after complete treatment	Treatment-related events not consistently separated from disease progression	Two combined cases and unadjusted treatment selection
Boström et al., 2009 (Boström et al., 2009)	Improved 6/20, stable 10/20, worsened 4/20	14/20 (70%) DSA-confirmed; six additional cases assessed intraoperatively or by MRI	3/20 residual; recurrence after DSA-confirmed cure not separately quantified	Early worsening 4/20; persistent decline 1/20; two CSF/arachnopathy reoperations	Seven patients received preoperative embolization
Lu et al., 2024 (Lu et al., 2024)	Follow-up mALS median 1; 16.7% unfavorable prognosis overall	Nidus subset 30.7%; surgery 60.8%, embolization 13.1%	Two delayed reconstitutions in full cohort; after partial treatment, acute/gradual deterioration 4.1%/year and 6.6%/year	Full cohort deterioration: surgery 14.7%, embolization 6.2%	Cervical-only cohort; combined sequence not separated
Yu et al., 2021 (Yu et al., 2021)	Annual deterioration declined from 32.5%/year to 9.3%/year	Nidus-type 29.3%; embolization-only 68/258 (26.4%); microsurgical exposure 120/205 (58.5%), including combined cases	Recanalization 73/219 (33.3%); after complete embolization 15/30 (50.0%); partial lesions: aneurysm 11.5%, proliferation 11.5%, spontaneous cure 1.6%	Permanent deterioration 11.2% overall; microsurgery 16.1%, embolization 5.6%	Microsurgical estimate includes 83 combined-treatment patients
Ushikoshi et al., 2000 (Ushikoshi et al., 2000)	Improved 3, stable 6, worsened 6	4/15 (27%)	11/15 residual; recurrence after documented cure not separated	One severe embolization-related paraparesis; five surgical deteriorations	Older techniques; timing and permanence incompletely reported
Lizana et al., 2022 (Lizana et al., 2022)	Two improved, one stable, one poor outcome	3/4 final angiographic obliteration	One residual lesion; case-level evolution reported	No standardized complication table	Four mixed-age cases; one complex staged pathway

Abbreviations: ASIA, American Spinal Injury Association; AVM, arteriovenous malformation; CSF, cerebrospinal fluid; DSA, digital subtraction angiography; IQR, interquartile range; mALS, modified Aminoff-Logue Scale; McCormick, modified McCormick Scale; MRA, magnetic resonance angiography; MRI, magnetic resonance imaging; mRS, modified Rankin Scale.

### 3.2 Treatment pathways, embolization intent and crossover reporting

Combined management was common but was not completely disaggregated. Rangel-Castilla reported perioperative embolization in 42% of predominantly surgical AVM cases, Boström reported preoperative embolization in 7/20 surgical patients, and Yu explicitly reported 83 combined-treatment patients. Lee reported two combined cases and one of four Lizana cases underwent staged embolization, partial surgery and additional embolization. None of the seven reports provided a reproducible count of unplanned conversion from embolization to surgery, distinct from a planned multimodality strategy. Accordingly, the crossover rate could not be estimated, and the combined cases were not treated as surgery-alone or embolization-alone. The treatment pathways, embolization intent, and crossover reporting are summarized in Table 5.

**Table 5:** Treatment pathways, embolization intent, and availability of crossover data. Patients who received combined treatment were not reassigned to the single-modality group.

Study	Embolization Alone	Surgery Alone	Planned Combined or Adjunctive Care	Intent and Crossover Reporting
Rangel-Castilla 2014	13%	Predominant pathway (86% surgical exposure)	Perioperative embolization in 42%; one radiosurgery case	Combined outcomes and unplanned crossover not separately reported
Lee et al., 2014	18 nidus cases	4 nidus cases	2 nidus cases	Combined outcomes limited; crossover not reported
Boström et al., 2009	None	20	Preoperative embolization in 7/20	Adjunctive embolization outcomes not separated
Lu et al., 2024	120 nidus cases	79 nidus cases	Not disaggregated	Intent and crossover not reported separately
Yu et al., 2021	258	122	83 planned combined cases	Partial embolization separated into shunt-reduction and palliative strategies; unplanned crossover not reported
Ushikoshi et al., 2000	5	10	Radiation adjunct in 4	Sequence and intent incompletely reported
Lizana et al., 2022	3	0	1 staged embolization-surgery-embolization pathway	Case-level intent reported; no unplanned crossover count

### 3.3 Risk of bias assessment

**Fig. 3:**
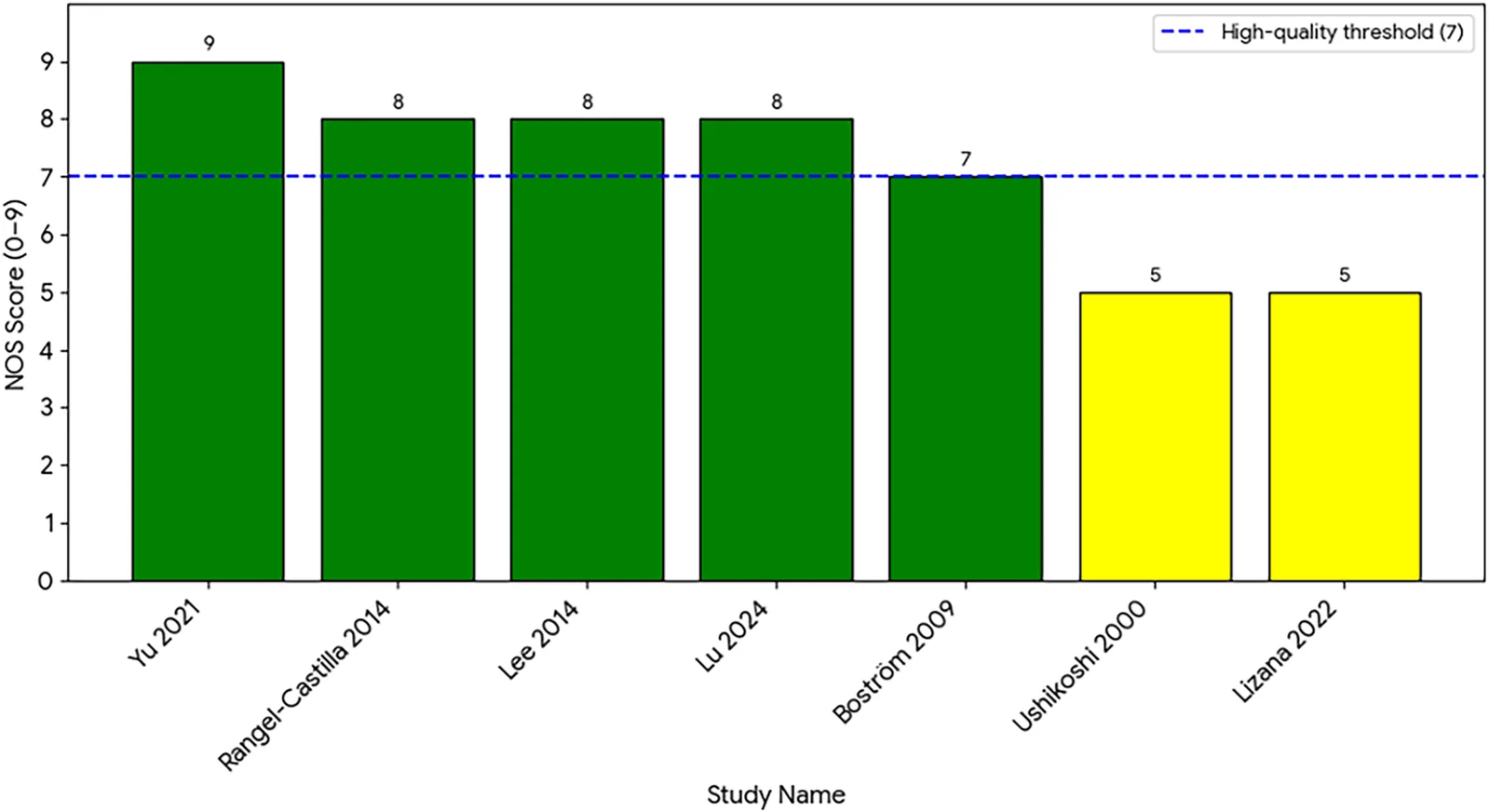
Newcastle-Ottawa Scale quality assessment scores for the seven included retrospective studies. A score of ≥7 (green) indicates high methodological quality. Yu et al. (2021) achieved the highest score (9/9). Ushikoshi et al. (2000) and Lizana et al. (2022) scored 5/9 because of limited comparability and incomplete outcome ascertainment.

The Newcastle-Ottawa Scale scores ranged from 5 to 9 (Table 3; Fig. 3). Four studies scored at least 7, whereas Ushikoshi et al. (Ushikoshi et al., 2000)and Lizana et al. (Lizana et al., 2022)scored 5 because of limited comparability and incomplete outcome ascertainment. These ratings do not remove the central risk of bias: no study randomized treatment, and most did not adequately adjust for lesion architecture, location, baseline severity, treatment intent, or combined treatment. Potential overlap between large referral center cohorts could not be evaluated. Therefore, comparative evidence remains vulnerable to confounding by indication, despite moderate or high NOS scores.

### 3.4 Radiographic endpoints after complete and partial treatment

**Fig. 4:**
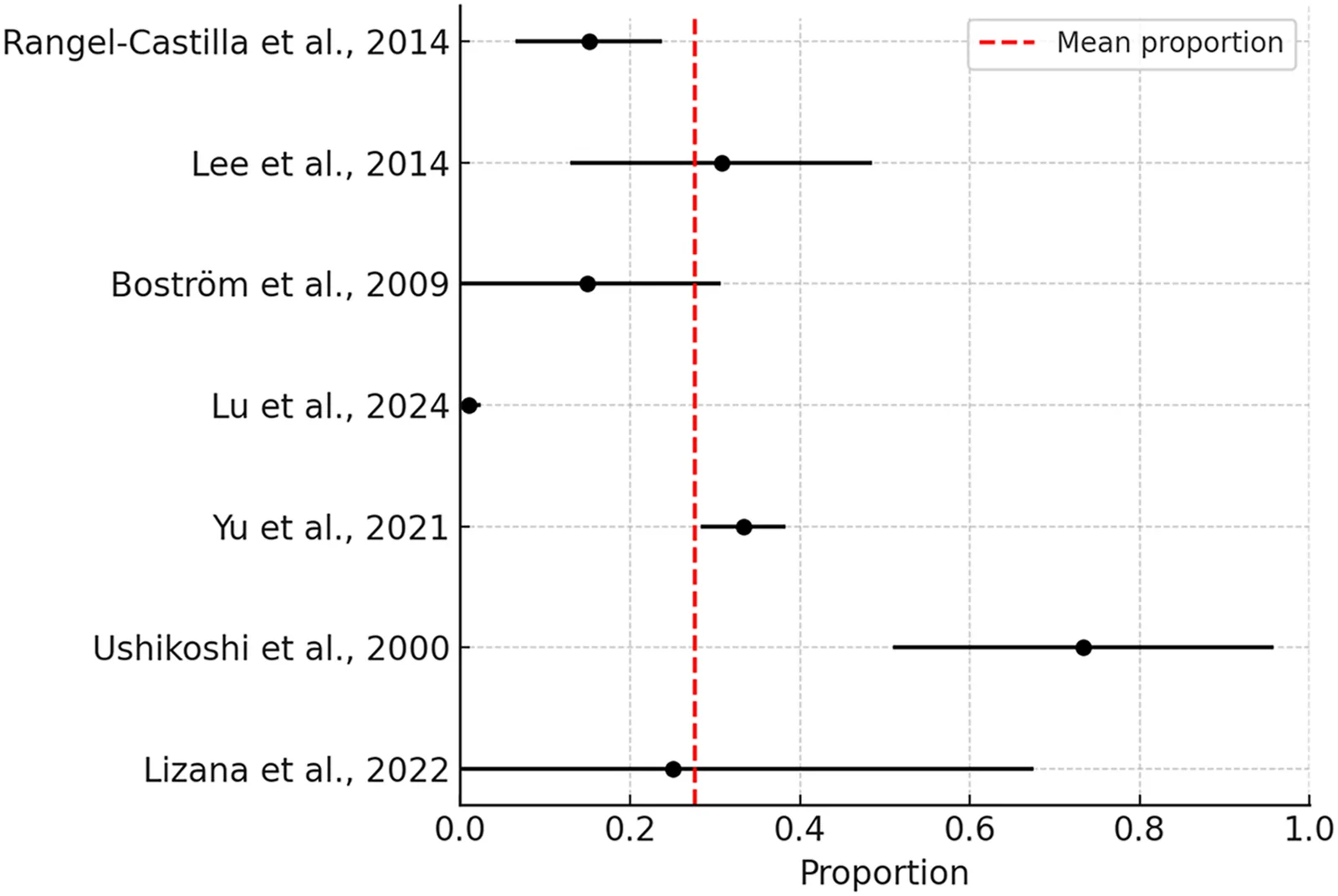
Descriptive study-level plot of reported residual or recurrent vascular shunts after treatment of Type II/III Si-AVMs. The endpoints were heterogeneous and should not be interpreted as a pooled recurrence rate after a confirmed angiographic cure.

Radiographic outcomes were reclassified using pre-specified definitions. The most informative disaggregated data were obtained by Yu et al. Among the 30 lesions considered completely obliterated after embolization and followed by DSA, 15 recanalized (50.0%). Of the 219 embolized lesions with follow-up DSA, 73 (33.3%) were recanalized. Among 191 partially embolized lesions, 22 (11.5%) developed a new aneurysm, 22 (11.5%) showed lesion proliferation, and 3 (1.6%) subsequently obliterated spontaneously (Yu et al., 2021). These outcomes should not be collapsed into a single failure rate, as recanalization after documented cure, persistence of a known residual shunt, and progression of a residual lesion are biologically and clinically distinct. In Lee et al., rebleeding was 0 after complete treatment compared with 2.9% per year after partial treatment; Lu et al. reported ongoing acute and gradual deterioration after partial treatment but not a radiographic recurrence rate specific to complete embolization (Lee et al., 2014; Lu et al., 2024). Therefore, Fig. 4 is a descriptive plot of reported residual or recurrent shunt proportions, not a pooled estimate of recurrence after a uniform angiographic cure. The distinction between complete and partial treatment outcomes is summarized in Table 6.

**Table 6:** Radiographic outcomes after complete treatment versus planned or known partial treatment. Residual disease, progression, recanalization, and recurrence were not considered interchangeable endpoints.

Study	After Documented Complete Treatment	After Partial or Residual Treatment	Interpretation
Yu et al., 2021	Recanalization after complete embolization: 15/30 (50.0%)	New aneurysm 22/191 (11.5%); proliferation 22/191 (11.5%); spontaneous obliteration 3/191 (1.6%)	Separates recanalization after cure from evolution of known residual lesions
Lee et al., 2014	Rebleeding 0 after complete treatment	Rebleeding 2.9%/year after partial treatment	Clinical durability reported; complete-embolization recurrence not isolated
Lu et al., 2024	Two delayed reconstitutions in full cohort: one after embolization and one after surgery	Acute deterioration 4.1%/year and gradual deterioration 6.6%/year after partial treatment	Clinical outcomes after partial treatment reported more clearly than radiographic recurrence
Boström et al., 2009	14/20 DSA-confirmed complete	3/20 residual	Preoperative embolization in 7/20 prevents a pure surgery-alone interpretation
Rangel-Castilla 2014/Ushikoshi et al., 2000	Not consistently separated	Residual or recurrent shunts reported as aggregate categories	Insufficient information to calculate recurrence after documented cure

### 3.5 Complete angiographic obliteration across treatment pathways

**Fig. 5:**
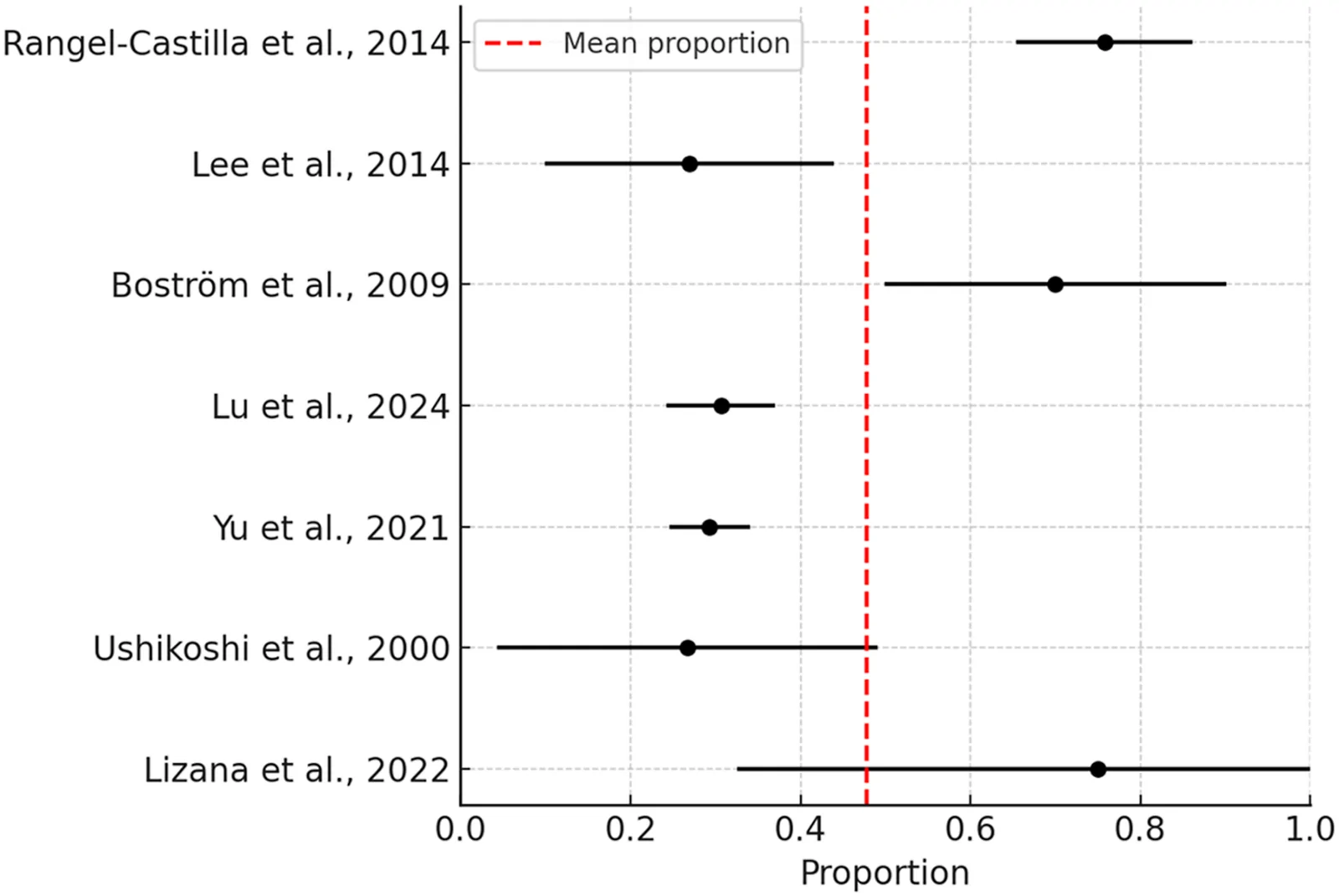
Descriptive study-level plot of complete angiographic obliteration after treatment of Type II/III Si-AVMs. Mixed treatment pathways, outcome definitions, and confirmation methods precluded a causal pooled comparison.

Complete obliteration varied according to the architecture, treatment intent, and confirmation method. In Yu et al., 68/258 (26.4%) embolization-only patients were cured, whereas 120/205 (58.5%) patients exposed to microsurgery were cured; the latter denominator included 83 combined-treatment patients and was not a surgery-alone estimate (Yu et al., 2021). In Boström et al., 14/20 (70%) Type II lesions were DSA-confirmed complete, while six additional cases were considered complete by intraoperative assessment or MRI (Boström et al., 2009). In the Lu nidus-type cervical subset, the reported complete obliteration rates were 60.8% after surgery and 13.1% after embolization (Lu et al., 2024). These unadjusted associations were strongly confounded: compact accessible lesions were more likely to be resected, whereas diffuse or metameric high-risk lesions were more likely to receive embolization, staged treatment, or palliation. Fig. 5 was retained as a descriptive study-level display only.

### 3.6 Functional improvement

**Fig. 6:**
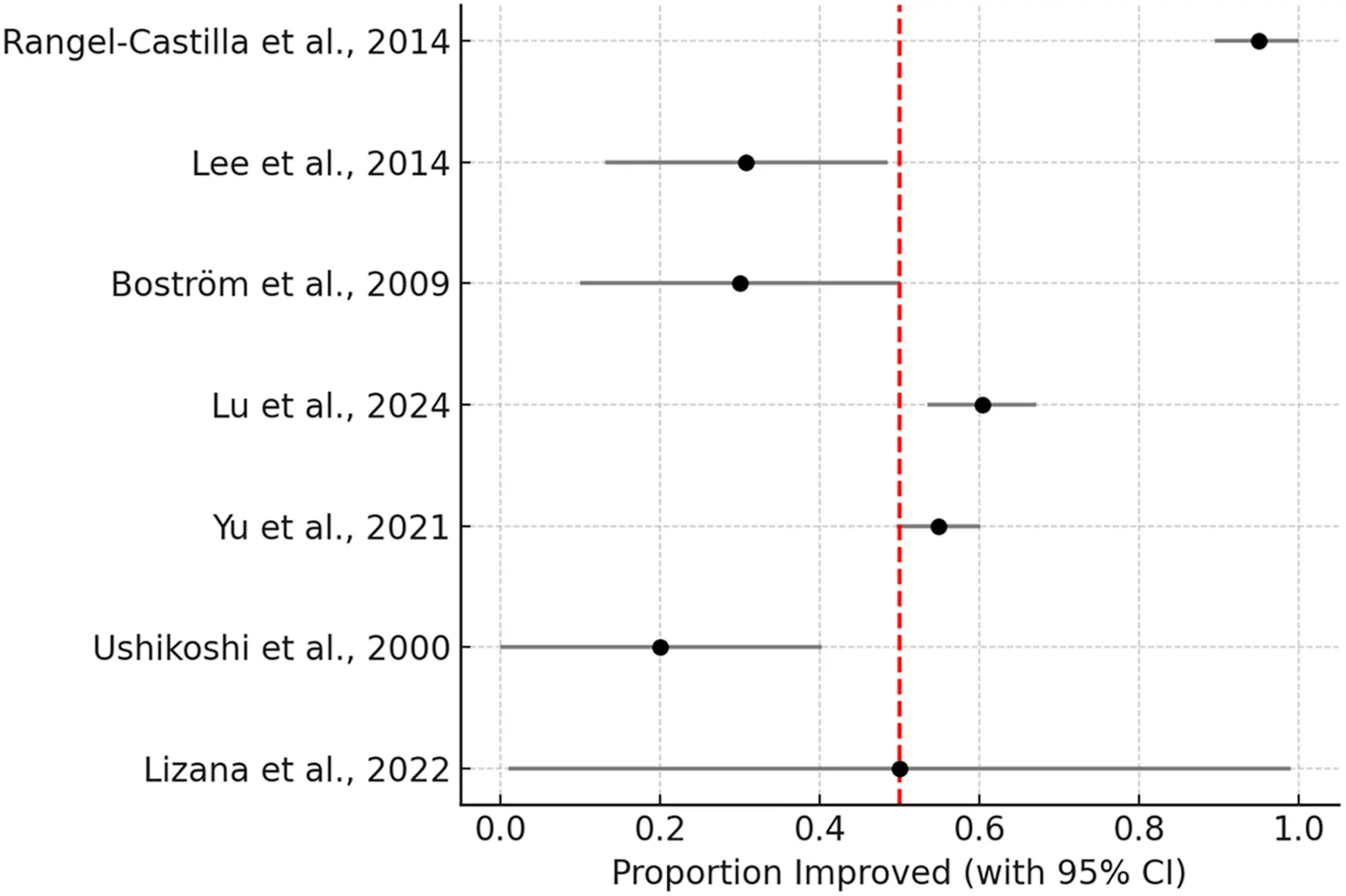
Descriptive study-level plot of functional improvement after treatment of Type II/III Si-AVMs. Outcome scales and improvement thresholds were not standardized across the studies.

Functional outcomes could not be reliably pooled by modality because the scales, time points, baseline severity, treatment intent, and combined-treatment classification differed. Lee et al. reported improvement in 8/26 (31%), stability in 12/26 (46%) and deterioration in 6/26 (23%) nidus-type lesions. Boström et al. reported 6/20 improved, 10/20 stable and 4/20 worse at long-term follow-up. (Lu et al., 2024) reported a median mALS improvement from 2 to 1 in cervical cohort and Yu et al. reported a reduction in annual deterioration after intervention. Fig. 6 displays the reported study-level improvement proportions without implying a common endpoint or treatment-specific causal effect.

### 3.7 Procedure-related complications

**Fig. 7:**
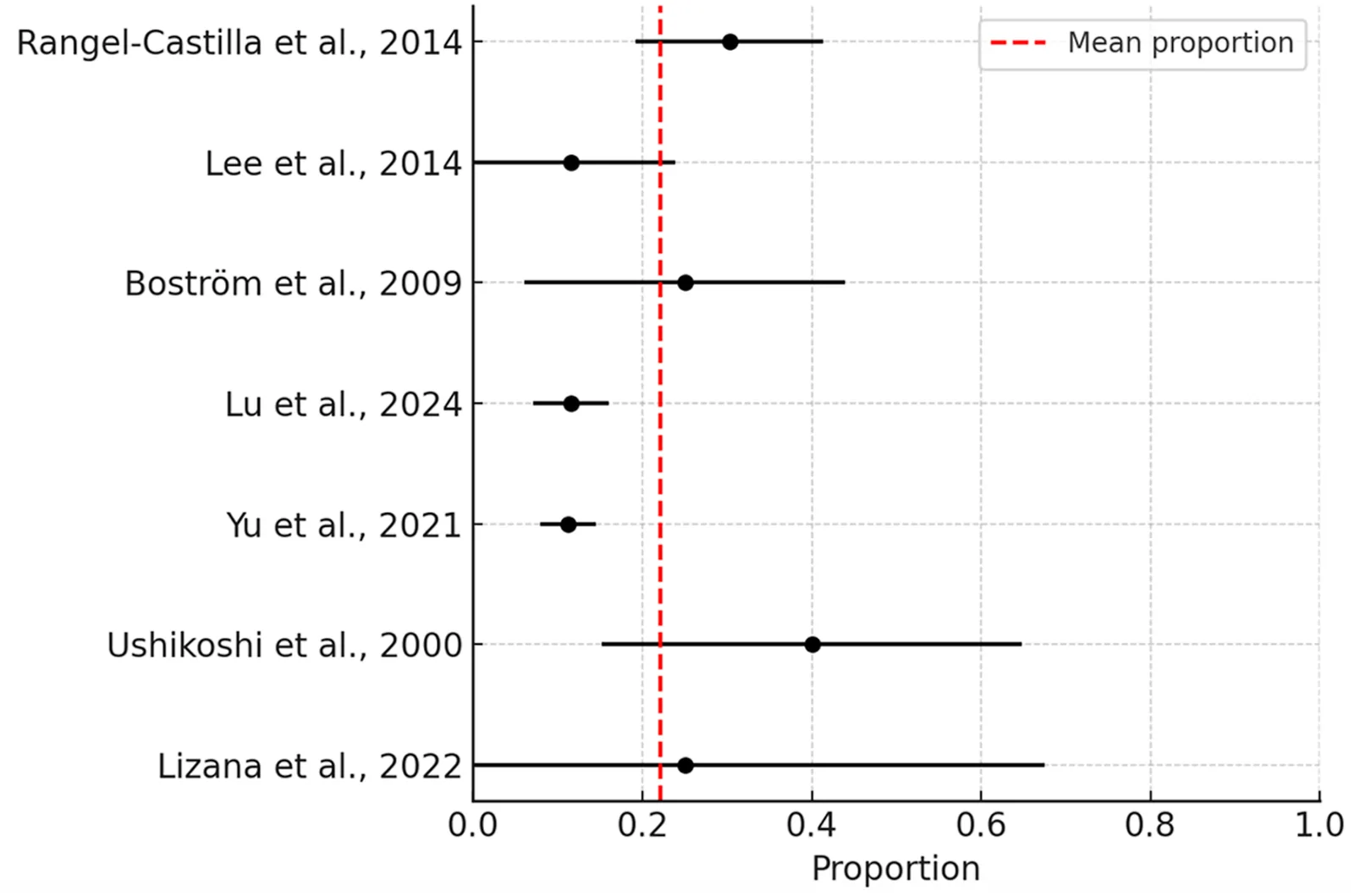
Descriptive study-level plot of reported procedure-related complications in Type II/III Si-AVM management. The definitions, timing, permanence, and treatment pathways varied across the studies.

The definitions of complications were heterogeneous. Where details were available, events were recoded as hemorrhagic, ischemic, thromboembolic, surgical, CSF-related, or radiation-related, and as acute or delayed and transient or permanent. Yu et al. reported permanent treatment-related deterioration in 11.2% overall, 16.1% after microsurgery and 5.6% after embolization (Yu et al., 2021). Boström et al. reported early neurological worsening in 4/20 (20%), persistent decline in 1/20 (5%) and two reoperations for arachnopathy or CSF leak (Boström et al., 2009). Ushikoshi et al. reported one severe embolization-related paraparesis and five surgical deteriorations but timing and permanence were not uniformly specified (Ushikoshi et al., 2000). Rangel-Castilla et al. reported an overall complication rate of 30.3% without consistent separation by pathway (Rangel-Castilla et al., 2014). Fig. 7 is a descriptive display; differences should not be interpreted as adjusted modality effects.

### 3.8 Lesion architecture, location and endovascular technique

However, subtype- and location-specific comparative data are sparse. (Boström et al., 2009) studied only Type II glomus AVMs, whereas (Lee et al., 2014) and (Yu et al., 2021) reported broader nidus-type categories that combined compact and diffuse lesions; (Lizana et al., 2022) described two compact and two diffuse cases and (Lu et al., 2024) was restricted to cervical lesions. (Yu et al., 2021)reported lower complete obliteration in metameric than nonmetameric lesions (18.1% vs 50.8%) but did not provide a complete Type II-versus-Type III or cervical-versus-thoracic/conus treatment comparison. Consequently, no objective pooled evidence has established that a particular modality is superior within each Takai subtype or spinal level. Embolic agent reporting was also insufficient for comparison: the studies spanned particles, coils, NBCA or Histoacryl, and evolving catheter techniques; (Yu et al., 2021) specifically reported that nonadhesive liquid embolic agents such as Onyx were not used in its participating centers. No included dataset supported an adjusted Onyx-versus-NBCA-versus-coil analysis.

### 3.9 Publication bias

Publication bias was not formally evaluated. With only seven studies, heterogeneous outcomes, unequal denominators, possible cohort overlap, and no common modality-specific effect estimate, a funnel plot or regression-based test would be statistically uninformative and potentially misleading. Therefore, the previously presented funnel plot was removed. Nevertheless, selective publications from high-volume centers remain possible.

### 3.10 Heterogeneity and temporal bias

The studies span 2000-2024, during which microsurgical visualization, intraoperative neuromonitoring, microcatheters, and embolic materials evolved substantially. Earlier reports used particles, coils, and adhesive agents such as NBCA or Histoacryl; later practice incorporated more selective catheterization and, at some centers, non-adhesive agents. However, agent, era, lesion architecture, and treatment intent were not jointly stratified; therefore, temporal or material-specific effects could not be estimated. The follow-up period ranged from months to 34 years, further complicating the recurrence comparisons.

## 4 Discussion

Type II/III Si-AVMs should not be reduced to a binary contest between surgery and endovascular embolization. The included reports described a spectrum of care, including surgery alone, embolization alone, neoadjuvant flow reduction, staged shunt reduction, targeted palliative embolization, planned combined treatment, adjunctive radiation, and observation. The unadjusted series associated microsurgical exposure with higher complete obliteration, but the same evidence showed strong selection by anatomy and intent. Therefore, the appropriate inference is not that one modality is globally superior, but that treatment durability and morbidity depend on lesion architecture, location, clinical state, and purpose of each procedure.

### 4.1 Extent of occlusion

Complete obliteration was most frequent in the selected surgical series and least frequent in the embolization-dominant nidus cohorts. However, the comparison was not treatment-randomized and was further distorted when combined-treatment patients were counted as surgical cases. In Yu et al., the 58.5% cure figure applied to all 205 patients exposed to microsurgery, including 83 who also underwent embolization, whereas 26.4% applied to 258 embolization-only patients (Yu et al., 2021). In a study by Boström et al., seven of 20 surgical patients underwent preoperative embolization (Boström et al., 2009). Thus, the available data support an association between microsurgical exposure and complete obliteration in selected lesions, not a standalone causal superiority estimate.

### 4.2 Multimodality treatment and embolization intent

Embolization has at least four distinct roles: attempted cure, preoperative flow reduction, staged reduction of shunt burden, and palliative targeting of aneurysms or other high-risk compartments. These roles cannot be combined without losing their clinical significance. Yu et al. separated partial embolization into shunt-reduction and palliative categories and reported no permanent treatment-related deterioration after palliative obliteration; the palliative strategy was directed toward hemorrhagic risk, whereas shunt reduction was more relevant to gradual myelopathy (Yu et al., 2021). Lee et al. observed no rebleeding after complete treatment but persistent risk after partial treatment (Lee et al., 2014) and Lu et al. documented continued clinical deterioration after partial treatment despite generally favorable overall outcomes (Lu et al., 2024). Therefore, planned partial embolization should be judged against its intended target and not automatically labeled as treatment failure.

### 4.3 Neurological improvement and functional outcomes

Functional improvement cannot be reliably assigned to one modality based on these data. Outcome scales (mALS, modified McCormick, ASIA, and mRS), thresholds, baseline severity, and follow-up intervals varied, and many combined cases were not separated. Across cohorts, stabilization was common, and the risk of deterioration generally declined after the intervention; however, selection and survivorship bias remained. Baseline neurological condition and hemorrhagic versus gradual presentation likely influence recovery as much as the treatment modality. The previously stated approximate 60% versus 35% comparison was removed because compatible treatment-specific denominators were unavailable.

### 4.4 Complications and morbidity considerations

Safety comparisons require equally careful interpretation. Yu et al. reported more permanent treatment-related deterioration after microsurgery than embolization (16.1% vs 5.6%) but microsurgical patients may have differed in lesion anatomy and therapeutic objective (Yu et al., 2021). Boström et al. reported 20% early versus 5% persistent worsening, illustrating why transient and permanent deficits must be separated (Boström et al., 2009). Endovascular complications include hemorrhage, ischemia or thromboembolism, and catheter- or embolysate-related injuries. Surgical complications include cord manipulation, infarction or hemorrhage, CSF leak, and arachnopathy. Radiosurgery carries the risk of delayed myelopathy. The source literature did not support a fully standardized complication meta-analysis.

### 4.5 Influence of lesion subtype and location

Objective subtype-specific evidence is limited. Boström et al. demonstrated that selected Type II glomus AVMs can be treated surgically with high DSA-confirmed obliteration but this does not establish superiority over embolization in comparable Type II lesions (Boström et al., 2009). Lee et al. (Lee et al., 2014)and Yu et al.(Yu et al., 2021) grouped lesions as nidus-type and diffuse Type III subgroup was not consistently separated. Yu et al. showed that metameric architecture was associated with lower complete obliteration (18.1% vs 50.8% in nonmetameric lesions), supporting the importance of architecture without proving a modality-specific effect (Yu et al., 2021). (Lu et al., 2024) provides cervical-only data, while conus and thoracic lesions were represented in other small series. No valid pooled cervical-versus-thoracic/conus or Type II-versus-Type III comparison was possible; therefore, treatment conclusions must remain lesion-specific and cautious.

### 4.6 Relationship to previous literature and modern endovascular practice

The revised interpretation aligns with the literature that treats embolization and surgery as complementary rather than mutually exclusive (Endo et al., 2016; Wilson et al., 2012; Yu et al., 2021; Ghobrial et al., 2018; Flores et al., 2017; Krings et al., 2009). Modern endovascular therapy can improve selectivity and may permit curative penetration in favorable lesions; however, the included studies did not provide agent-adjusted comparisons. The absence of Onyx in the large Yu cohort and inconsistent reporting of NBCA or Histoacryl, particles, and coils make any claim of material-specific superiority unsupported. These limitations are clinically important because outcomes from early-2000s techniques should not be assumed to represent contemporary endovascular practice.

### 4.7 Role of radiosurgery

Radiosurgery was not formally pooled because the evidence consisted of small, heterogeneous series with different lesion types, doses, fractionation schemes, and follow-ups. Fractionated radiotherapy and SRS reports generally described neurological stability or nidus reduction but complete obliteration was modest: Hida et al. reported nidus reduction without complete cure in 10 cases (Hida et al., 2003); Mori et al. reported two angiographic cures (Mori et al., 2018); small series by Rashad et al. and Potharaju et al. reported 0-25% obliteration (Rashad et al., 2017; Potharaju et al., 2014); and Kalani et al. reported 19% complete obliteration in 37 patients (Kalani et al., 2016). These findings define a niche rather than an established equivalent of surgery or embolization. SRS may be considered for selected deep, diffuse, or surgically inaccessible lesions when other approaches cannot safely address the nidus; however, latency leaves a period of ongoing hemorrhage risk, and delayed radiation myelopathy remains a concern. Current evidence is insufficient to establish an optimal dose, identify a responsive subtype, or support direct comparative efficacy claims (Zhan et al., 2019; Wolkov and Bagshaw, 1988; Hida et al., 2003; Mori et al., 2018; Rashad et al., 2017; Potharaju et al., 2014; Kalani et al., 2016; Suzuki et al., 2023).

### 4.8 Recommendations and management approach

Given the limitations of the evidence, management should follow a multidisciplinary lesion-specific algorithm. For a compact, superficial Type II lesion with a feasible dissection plane, microsurgery, often after selective preoperative embolization, may offer a durable cure, but the evidence is observational. Embolization may be used with curative intent when safe nidus penetration is possible, such as staged flow reduction, to secure aneurysms or other high-risk compartments, or as palliation. Diffuse Type III or metameric and surgically inaccessible lesions may require staged or combined treatment, observation, or selected radiosurgery. Long-term DSA or MRA surveillance is necessary after complete embolization and after partial treatment. The available data do not support a universal hierarchy of modalities.

### 4.9 Limitations

This study had several limitations. First, all seven reports were retrospective observational cohorts or case series; treatment assignment was non-random and incompletely adjusted for architecture, location, baseline severity, and treatment intent. Second, surgery-alone, embolization-alone, and combined pathways were inconsistently separated, and no study reported a reproducible unplanned crossover rate distinct from planned multimodal care. Third, complete obliteration, residual disease, recurrence, recanalization, and progression were not uniformly defined in the source literature, and functional and complication outcomes used different scales, thresholds, and follow-up times. Fourth, subtype-, location-, and embolic agent-specific data were too sparse for reliable pooled analyses. Fifth, mixed-age and mixed-lesion cohorts were included when relevant nidus-type data were extractable; the reported maximum of 672 observations should not be interpreted as 672 confirmed, unique, adult patients.

Sixth, overlap between the large Yu and Lu referral center cohorts could not be excluded. Seventh, the follow-up period ranged from months to 34 years, limiting comparisons of late recurrence. Eighth, the restriction of English-language publications and exclusion of series with fewer than three patients may have introduced selection bias. Ninth, no included study used standardized patient-reported outcome measures. Finally, formal publication bias testing was not appropriate for the seven heterogeneous studies, although selective reporting from high-volume centers remains possible. Therefore, quantitative displays and all treatment comparisons should be considered hypothesis-generating rather than practice-defining.

## 5 Conclusion

This systematic review supports a complementary lesion-specific approach to Type II/III Si-AVMs. Microsurgical exposure was associated with higher complete obliteration in selected cohorts, while embolization was associated with lower reported permanent morbidity in same-cohort data and served curative, staged, neoadjuvant, or palliative roles. Planned partial embolization must be evaluated according to its intended target, and recanalization after complete treatment must be separated from residual or progressive disease. Because all evidence was retrospective, heterogeneous, and potentially overlapping, no modality superiority or subtype- or location-specific treatment ranking could be established. Prospective multicenter registries should record the architecture, location, treatment sequence and intent, embolic agent, DSA-defined endpoints, standardized transient and permanent complications, and long-term functional outcomes.

## Availability of data and materials

All data generated or analyzed in this study are included in this article. Further inquiries can be directed to the corresponding author.

## Funding

This study did not receive any funding or financial support.

## Declaration of interests

The authors declare that they have no known competing financial interests or personal relationships that could have appeared to influence the work reported in this paper. No external funding, grants, or industry support was received for the conduction of the study, analysis, or preparation of the manuscript. The authors have not received honoraria, consulting fees, stocks, or other forms of compensation from entities with an interest in the subject matter discussed. All authors had full access to the data and take responsibility for the integrity and accuracy of the analysis. The manuscript has not been published previously and is not under consideration elsewhere.

## References

[bib1] Akgun M.Y., Ucer M., Tanriverdi O. (2019). Spinal vascular malformations: treatment and outcomes. World Neurosurg..

[bib2] Bergstrand A., Höök O., Lidvall H. (1964). Vascular malformations of spinal cord. Acta Neurol. Scand..

[bib3] Boström A., Krings T., Hans F.J., Schramm J., Thron A.K., Gilsbach J.M. (2009). Spinal glomus-type arteriovenous malformations: microsurgical treatment in 20 cases. J. Neurosurg. Spine.

[bib4] Endo T., Endo H., Sato K., Matsumoto Y., Tominaga T. (2016). Surgical and endovascular treatment for spinal arteriovenous malformations. Neurol. Med.-Chir..

[bib5] Flores B.C., Klinger D.R., White J.A., Batjer H.H. (2017). Spinal vascular malformations: treatment strategies and outcome. Neurosurg. Rev..

[bib6] Ghobrial G.M., Liounakos J., Starke R.M., Levi A.D. (2018). Surgical treatment of vascular intramedullary spinal cord lesions. Cureus.

[bib7] Gross B.A., Du R. (2013). Spinal glomus (Type II) arteriovenous malformations: a pooled analysis of hemorrhage risk and results of intervention. Neurosurgery.

[bib8] Hida K., Shirato H., Isu T., Seki T., Onimaru R., Aoyama H., Ushikoshi S., Miyasaka K., Iwasaki Y. (2003). Focal fractionated radiotherapy for intramedullary spinal arteriovenous malformations: 10-year experience. J. Neurosurg. Spine.

[bib9] Kalani M.Y.S., Choudhri O., Gibbs I.C. (2016). Stereotactic radiosurgery for intramedullary spinal arteriovenous malformations. J. Clin. Neurosci..

[bib10] Kona M.P., Buch K., Singh J., Rohatgi S. (2021). Spinal vascular shunts: a patterned approach. AJNR Am J Neuroradiol.

[bib11] Krings T., Geibprasert S., terBrugge K.G. (2009). Endovascular management of spinal vascular malformations. Neurosurg. Rev..

[bib12] Lee Y.J., Terbrugge K.G., Saliou G., Krings T. (2014). Clinical features and outcomes of spinal cord arteriovenous malformations. Stroke.

[bib13] Lizana J., Aliaga N., Marani W., Escribano A., Montemurro N. (2022). Spinal vascular shunts: single-center series and review of literature of their classification. Neurol. Int..

[bib14] Long H.A., French D.P., Brooks J.M. (2020). Optimising the value of Critical Appraisal skills programme (CASP) tool for quality appraisal in qualitative evidence synthesis. Res. Methods Med. Health Sci..

[bib15] Lu H.H., Lin W.C., Chien J.C., Wu C.C., Hsu Y.H., Chen W.H. (2024). The natural course, treatment outcomes and long-term prognosis of cervical spinal cord arteriovenous shunts. J. Neurosurg..

[bib16] Mamaril-Davis J., Aguilar-Salinas P., Avila M.J., Dumont T., Avery M.B. (2023). Recurrence rates following treatment of spinal vascular malformations: a systematic review and meta-analysis. World Neurosurg..

[bib17] Minozzi S., Cinquini M., Gianola S., Gonzalez-Lorenzo M., Banzi R. (2020). The revised Cochrane risk-of-bias tool for randomised trials (RoB 2) showed low inter-rater reliability and challenges in its application. J. Clin. Epidemiol..

[bib18] Mori Y., Hashizume C., Tsugawa T., Kato S., Shibamoto Y. (2018). Stereotactic radiotherapy for intramedullary spinal arteriovenous malformations. Cureus.

[bib19] Parums D.V. (2021). Editorial: review articles, systematic reviews, meta-analysis and updated Preferred Reporting Items for Systematic Reviews and Meta-Analyses (PRISMA) 2020 guidelines. Med. Sci. Monit..

[bib20] Potharaju M., John R., Venkataraman M., Gopalakrishna K., Subramanian B. (2014). Stereotactic radiosurgery results in three cases of intramedullary spinal cord arteriovenous malformations. Spine J..

[bib21] Rangel-Castilla L., Klucznik R., Diaz O., Kalani M.Y.S., Spetzler R.F. (2014). Contemporary management of spinal AVFs and AVMs: lessons learned from 110 cases. Neurosurg. Focus.

[bib22] Rashad S., Endo T., Ogawa Y. (2017). Stereotactic radiosurgery as a feasible treatment for intramedullary spinal arteriovenous malformations: a single-center observation. Neurosurg. Rev..

[bib23] Rosenblum B., Oldfield E.H., Doppman J.L., Di Chiro G. (1987). Spinal arteriovenous malformations: comparison of dural arteriovenous fistulas and intradural AVMs in 81 patients. J. Neurosurg..

[bib24] Spetzler R.F., Detwiler P.W., Riina H.A., Porter R.W. (2002). Modified classification of spinal cord vascular lesions. J. Neurosurg..

[bib25] Suzuki T., Kagawa K., Sato K. (2023). CyberKnife radiosurgery for spinal intramedullary arteriovenous malformations: a single-center experience. World Neurosurg..

[bib26] Takai K. (2017). Spinal arteriovenous shunts: angioarchitecture and historical changes in classification. Neurol. Med.-Chir..

[bib27] Ushikoshi S., Hida K., Kikuchi Y., Iwasaki Y., Miyasaka K., Abe H. (2000). Treatment of intramedullary arteriovenous malformations of spinal cord. Intervent Neuroradiol..

[bib28] Velat G.J., Chang S.W., Abla A.A., Albuquerque F.C., McDougall C.G., Spetzler R.F. (2012). Microsurgical management of glomus spinal arteriovenous malformations: pial resection technique. J. Neurosurg. Spine.

[bib29] Wilson D.A., Abla A.A., Uschold T.D., McDougall C.G., Albuquerque F.C., Spetzler R.F. (2012). Multimodality treatment of conus medullaris arteriovenous malformations: 2 decades of experience with combined endovascular and microsurgical treatments. Neurosurgery.

[bib30] Wolkov H.B., Bagshaw M.A. (1988). Conventional radiation therapy in management of arteriovenous malformations of central nervous system. Int. J. Radiat. Oncol. Biol. Phys..

[bib31] Yu J.X., Xu K., Sun H. (2021). The efficacy and deficiency of contemporary treatment for spinal cord arteriovenous shunts. Brain.

[bib32] Zhan P.L., Jahromi B.S., Kruser T.J., Potts M.B. (2019). Stereotactic radiosurgery and fractionated radiotherapy for spinal arteriovenous malformations: a systematic review of literature. J. Clin. Neurosci..

